# T113. THE LINK BETWEEN BLUNTED AFFECT AND SUICIDE IN SCHIZOPHRENIA: A SYSTEMATIC REVIEW

**DOI:** 10.1093/schbul/sby016.389

**Published:** 2018-04-01

**Authors:** Markella Grigoriou, Rachel Upthegrove, Lisa Bortolotti

**Affiliations:** University of Birmingham

## Abstract

**Background:**

The lifetime risk of suicide and suicide attempt in patients with schizophrenia are 5% and 25%–50%, respectively. Understanding the suicide risk factors is of great significance in research and clinical practice. The current systematic review is the first attempt to examine and demonstrate the associations between the core negative symptom of blunted affect and suicide in people with schizophrenia. We believe this review may have important implications for suicide epidemiology and helps us improve prevention tools.

**Methods:**

A comprehensive search strategy using PRISAMA guidelines was used to identify potential studies and data that met inclusion criteria. We searched original studies published since 2016 via MEDLINE (R) from 1946 to February 2016, EMBASE from 1947 to February 2016, and PsychINFO from 1806 to February 2016. Inclusion criteria were met if an article reported any kind of correlation between negative symptoms and suicide ideation, attempted suicide or completed suicide in patients with schizophrenia. The used search terms were: schizophreni* AND suicid* AND negative symptom* OR affective symptom* OR expressed emotion* OR emotional internal*. Studies with original data related to the blunted affect and suicide in schizophrenia were examined by manual reviewing.

**Results:**

The initial search found 878 papers about negative symptoms and suicidal behaviour. From those only 12 papers fulfilled the inclusion criteria. Eight of twelve eligible papers found a positive association between blunted affect and suicide in schizophrenia indicating the link between social isolation and blunted affect with suicide (p<0.018), the impact blunted affect has on completed suicides on female population with schizophrenia (p<0.034) and the link between blunted affect and suicide in the stage of hospital admission (p<0.001). Two of twelve papers report no significance between blunted affect and suicide. One paper shows blunted affect did not have direct relation with suicide but can lead to the development of a suicidal behaviour. The last paper demonstrates blunted affect is important as suicide risk factor in schizoaffective disorder only.

**Discussion:**

Based on the best available data, our results demonstrate a challenging link between blunted affect or related emotional disturbances and suicide in schizophrenia. Despite major suicide risk factors such as hopelessness, positive symptoms and depression, blunted affect is another factor we need to consider as it relates to social engagement and emotion regulation which are essential elements for eliminating suicides and improve interventions in psychiatry. Our outcomes may help with future development of preventive strategies and tools to combat suicide but also with gaining better understanding around what determines suicidal behaviour in schizophrenia.

